# When A Combination of Nudges Decreases Sustainable Food Choices Out-of-Home—The Example of Food Decoys and Descriptive Name Labels

**DOI:** 10.3390/foods9050557

**Published:** 2020-05-02

**Authors:** Pascal Ohlhausen, Nina Langen

**Affiliations:** Department Education for Sustainable Nutrition and Food Science, Institute of Vocational Education and Work Studies, Technische Universität Berlin, 10587 Berlin, Germany; nina.langen@tu-berlin.de

**Keywords:** descriptive name labels, out-of-home, catering, sustainable nutrition, food, nudge, decoy

## Abstract

This paper reports results from three consecutive studies focusing on the comparison of the effectiveness of different nudges and their combinations to increase sustainable food choices out of the home. The nudges compared are the use of descriptive name labels (DNLs) for the most sustainable dish of a choice set (menu) and the decoy effect (DE), created by adding a less attractive decoy dish to a more attractive target dish with the goal of increasing the choice frequency of the target dish. In the literature, both nudges have been found to influence consumers’ choices. In the first study, six category names of sustainability indicators were deduced from a focus group. These were tested with 100 students to identify the most attractive DNLs. Study II, a randomized choice study (*n* = 420), tested the DE, the DNLs and a combination of the DNLs and the DE used on four different dishes in a university canteen. In study III, 820 guests of a business canteen voted during four weeks for the special meals of the following week (identical to the four choice sets displayed in study II). Results indicate that the combination of DNLs and the DE is not recommended for fostering sustainable food choices. Pure DNLs were more efficient in increasing the choice frequency of the more sustainable meal, whereas the decoy effect resulted in decreased choice frequencies. Regional and sustainable DNLs were favoured by consumers.

## 1. Introduction

The out-of-home catering (OOHC) sector is a rapidly growing market in Germany with growth rates of about three to four percent per year [1,2,3,4]. Due to rising mobility and urbanization, increasing shares of single-person households, higher incomes and time pressure, the amount of meals consumed at home is constantly decreasing [5,6,7]. Despite consumers’ interest in the topics of sustainability or health [8,9], a considerable share of their food choices remain unsustainable and unhealthy [10,11,12,13]. One of the problems for consumers is procrastination (i.e., having an intention but failing to realise it) [14,15,16]. One possible approach to make customers’ food choices more sustainable is to nudge them, and thereby stimulate, facilitate and encourage the sector’s transformation towards sustainable development. The concept of nudging was first defined by Thaler and Sunstein [17] (p. 6): “A nudge, […] is any aspect of the choice architecture that alters people’s behaviour in a predictable way without forbidding any options or significantly changing their economic incentives. To count as a mere nudge, the intervention must be easy and cheap to avoid.” From this point on, the literature has focused on implementing and assessing nudges in several disciplines, e.g., tax and health policy, old-age provision and environmental protection [18,19,20,21,22,23]. In the sector of nutrition, several systematic reviews and meta-analyses have been conducted [24,25,26,27,28,29]. A meta-analysis by Arno and Thomas [29], for example, included 42 studies and revealed an increase in healthy food choices or decrease in energy intake of 15.3% when respective nudges were present. Besides laws and guidelines, nudges in the sector of nutrition can be used to further improve sustainability in the food sector, combat overweight as well as obesity and support the healthcare system without limiting consumers’ wish for freedom of choice [8,29,30]. To avoid being used as a fraudulent marketing instrument, nudges should be transparent, never misleading, easy to opt-out of, consistent with people’s values, improve the welfare of those being nudged and not violate individual rights [31,32]. Nudges in the nutrition sector range from choice-architecture-based nudges (also called system-1 nudges), such as changes in menu design or counter position, to information-based nudges (also called system-2 nudges), e.g., labels or additional nutritional information [20,33,34].

Employing the concept of nudging, this paper looks at consumers’ food choices in education and business catering settings with the goal of fostering sustainable OOHC transformation. 

To date, very little research has aimed at combining and comparing different nudging interventions in OOHC and surveyed their effects in simultaneous use [24,26,27]. Therefore, we opted to close this gap by combining and comparing two nudging interventions with the aim of increasing choice frequencies of sustainable dishes in OOHC. The nudges we combine and compare are (i) descriptive name labels (DNL), commonly referred to as food names, and (ii) the attraction effect or decoy effect (DE). The decoy effect in general, e.g., [35,36,37,38,39,40], and particularly descriptive name labels, e.g., [34,41,42,43,44,45], have been found to influence consumers’ perceptions and choices of goods. 

Most recent labelling studies have focused on food labels such as nutrition, health or warning labels and not on food names [41,46,47,48,49]. Nevertheless, DNLs are already used in restaurants and canteens, and the literature states increased choice frequency, higher taste ratings and more positive attitudes towards labelled dishes [41,42,43]. For preadolescent children, DNLs were used to boost vegetable and fruit intake [44]. The spectrum of name categories ranges from geographic DNLs (e.g., Southwestern), sensory DNLs (e.g., savory), nostalgic DNLs (e.g., grandma’s) to heroic DNLs (e.g., for superheroes) [41,42,44]. 

The decoy effect was first described as a concept of asymmetrically dominated alternatives by Huber et al. [50]. A decoy is an alternative added to a choice set in order to alter the relative attractiveness of the other alternatives in the choice set. The addition of asymmetrically dominated alternatives called decoys increases the proportion of choices in favour of the target. In supermarkets, for example, the decoy effect can be used to enhance chocolate bar sales by placing a similar but slightly inferior chocolate bar next to the target product [51]. “Inferior” relates to the price or other quality attributes of the target product. Hence, the decoy effect can be used to push customers in the direction of buying a certain target product by showing a slightly worse alternative to the target product [50,52]. In the food sector, a choice study found evidence for the attraction effect when choosing dishes that display their calorie ratings [53].

The research aim is to assess the effectiveness of a combination of the decoy effect (DE) and descriptive name labels to increase consumers’ choice of sustainable dishes. Hence, it is important to compare whether using a combination of these two nudging approaches is more effective in fostering sustainable food choice than the isolated nudging approaches on their own. The base of the studies was formed by a list of commonly offered OOHC dishes in Germany. Building on these dishes, the objective of study I is to find the best DNL wordings for six different DNL categories, which were derived by a focus group and literature survey, via a pre-test with students. The best DNLs per category are used in the choice experiment in study II. The objective of study II is to assess the effectiveness of DNLs in combination with the DE versus their single nudging variants in a university canteen setting. To reveal possible differences between settings, study III tests the combination of DNLs and the DE in a business canteen. For an overview of these studies, see Table 1.

## 2. Study I

### 2.1. Study I—Design

The first step was to define the dishes used as sustainable target dishes in the study. We, therefore, applied a tool developed and described by Engelmann et al. [54]. The tool is able to determine and compare the environmental and health impacts of dishes based on various criteria describing the four dimensions of sustainability: ecology, society, economy and health [54]. These criteria are, for example, the material footprint, carbon footprint, water demand, floor space demand, fair trade standards, animal welfare considerations, energy content, fibre content, fat content, carbohydrate content, sugar and salt content. Based on this assessment tool, we selected eight different commonly offered and frequently eaten OOHC meals with similar sales data (for more information see Study II), but with slightly higher health and sustainability ratings for the target dishes in comparison to the competitor dishes (see Table 2). 

To get more information about OOHC customers’ sustainability preferences, we conducted a focus group in February 2016. The seven selected customers were between 20 and 60 years old and ate several times per week in OOHC canteens, making them experienced and involved participants. The most interesting question for the study regarded their desired and undesired indicators for eating meals out-of-home. Therefore, the focus group brainstormed relevant aspects first and then discussed the desirability of each indicator for OOHC. To avoid biasing participants’ brainstorming process, no list of possible aspects was provided. Participants were free to mention any aspects they considered relevant. The 18 mentioned aspects and their desirability for meal characteristics are displayed in Table 3.

To combine dishes with different sustainable DNLs, different DNL categories were distinguished. The 18 aspects derived and discussed by the focus group were grouped into five sustainability categories for OOHC: regional (included the following aspects: regional, freshness, carbon footprint, ecological footprint, resource input) [41,42,56,57,58], seasonal (included the following aspects: seasonal, freshness, carbon footprint, ecological footprint, resource input) [56], organic (included the following aspects: free of, pesticide-free, animal welfare, carbon footprint, ecological footprint, additives, resource input, organic, free of genetic modifications) [59], sustainable (included the following aspects: animal welfare, carbon footprint, ecological footprint, resource input, fair trade, free of genetic modifications) [59] and healthy (included the following aspects: free of, pesticide-free, freshness, hygiene, additives, percentage of animal products, sugar, nutritional information, free of genetic modifications, portion size) [48,60]. These five categories were used as descriptive name label categories in studies I–III, described below.

In a following step, we screened menus from restaurants, canteens, cafés, bistros or snack bars as well as the literature [41,42] for insights into the great variety of possible DNLs for each category. It became clear that many DNLs describe traditional product and process characteristics. These were not identified during the focus group. Therefore, we added a sixth category to be used in studies I-III: the traditional name category.

A quantitative study was conducted in spring 2016 with students at TU Berlin. Students (*n* = 100) were asked to indicate their preferred name labels for several food name categories displayed in a list. The question posed in the questionnaire was ‘Imagine: It’s lunchtime and you’e standing hungrily in the canteen in front of the menu. Which of the following phrases appeals to you most?’. The idea was to select the best DNLs per category for later application in the choice experiment of study II and to sort out unappealing DNLs. This procedure was recommended in Wansink et al. [41] and ensures that the DNLs used in the following studies are of relevance for consumers [41,42].

Each respondent was asked to answer two of a total of five questionnaires. These five questionnaire variants included one of our four target dishes for study II or the neutral “dish” variant. Each questionnaire included all six DNL categories (traditional, regional, seasonal, organic, sustainable and healthy) with all available DNLs applied to the dish, as can be seen in Table 4 for the neutral variant.

We used five different questionnaire variants to minimize the possibility that one DNL is preferred when applied to one specific dish but disliked on other dishes by the OOHC guests. The respondents were only to answer two out of the five questionnaires due to the possibility of a priming effect. That is, it was necessary to avoid that students kept selecting the same DNLs in all five questionnaires without considering the specific combination of dishes and DNLs.

### 2.2. Study I—Results

Table 4 displays the results of Study I. For the traditional DNL category, the descriptive name “(Dish), traditional style” prevailed, with 63.5 percent of all votes. The choice results in the regional DNL category showed the DNL “(Dish) from regional agriculture” as the winner, with 28.5 percent of all votes. This DNL was modified for fish dishes, where the label “(Dish) from regional fishery” was used. Within the seasonal DNL category, the respondents clearly preferred the descriptive name “(Dish) with seasonal ingredients”, with 64 percent of all votes. As expected, the descriptive name “Organic (Dish)” earned 35.5 percent of all votes and was the clear winner in the organic DNL category. By a narrow margin, the descriptive name “(Dish) from sustainable agriculture" prevailed in the sustainable DNL category, with 22.5 percent of all votes. The equivalent “(Dish) from sustainable fisheries” was used for the fish dish. In the healthy DNL category, the descriptive names “(Dish), low-energy prepared” as well as “Low energy (Dish)” received the most approval, with 24.5 and 19 percent of the votes. The implementation of these DNLs in OOHC is problematic due to European health claim regulations [61]. Therefore, the option “(Dish) for light pleasure” with 17 percent of votes was used as a healthy DNL in the later studies. According to European health claim regulations, food may only be designated as low in energy if the product contains no more than 40 kcal per 100 g for solid goods and no more than 20 kcal per 100 ml for liquid goods [61]. These extremely low limit values cannot be complied with by the majority of OOHC meals. However, it is possible to use the DNL "(Dish) for light pleasure" or “Light (Dish)” in the OOHC. Here, the regulations require the same conditions that are applied to "reduced" dishes. "Energy-reduced" dishes must have a reduced caloric value of at least 30 percent compared to the common preparation of the dish [61].

## 3. Study II

### 3.1. Study II—Hypotheses

The overall goal of study II was to use both the decoy effect and DNL nudges to evaluate their combined impact on food choices in canteens. Currently, no published articles or other scientific studies on the topic exist. To close this gap, we developed the following five hypotheses:

- H1: “Consumers prefer meals with DNLs.”

To test this hypothesis, we assessed whether target dishes with the single use of descriptive name labels were chosen more often than target dishes without the use of this nudge. This was necessary to replicate the effect of DNLs, as stated in the literature [41,42,43,44,45], in our OOHC setting.

- H2: “Using the DE increases the choice frequency of the target dish.”

For the second hypothesis, we tested the decoy effect on target dishes compared to target dishes without this nudge to illustrate the general impact of the decoy effect [35,36,37,38,50,62] and its applicability in the OOHC setting.

- H3: “Consumers prefer those dishes promoted via the combination of both nudges in comparison to their common dish counterparts.”

Hypothesis 3 aimed to answer the general research question and illustrates the general impact of the combination of DNLs and the DE in OOHC settings. Therefore, target dishes with the applied nudge combination of DNLs and the DE were compared to common target dishes (target dishes without any nudges).

- H4: “The combination of DNLs and the DE leads to more sustainable choices than the isolated effects of the single nudges.”

The fourth hypothesis tested the impact of the combination of the two effects on consumer choices in comparison to meals with only DNLs or only the DE. The goal of this hypothesis was to avoid the use of inefficient nudge variants in OOHC.

- H5: “There is a significant difference between the effectiveness of different DNL categories and commonly named dishes in fostering sustainable meal choices.”

Recent studies showed a trend for regional-sourced, organic, as well as sustainable OOHC dishes in Germany [57]. The last hypothesis assessed which of the DNL categories are favoured over commonly named dishes. For this reason, we checked for significant differences between the DNLs and their common variants as well as between the single DNL categories.

From a theoretical point of view, we would expect both hypothesis 1 and hypothesis 2 to be supported. In this case, the literature of DNLs [41,42,43,44,45] and DE [35,36,37,38,40,50,62] would be consistent with the study results of this work on sustainable OOHC food. Based on the combination of the two nudges, we also expect to find support for hypothesis 3. Hypothesis 4 has never been tested before [24,26,27]. However, the sales and marketing effects stated in the literature on the pure DE [35,36,37,38,50,62] and the pure DNLs [41,42,43,44,45] could complement each other and further increase the sales of sustainable OOHC dishes. Therefore, we expect to find support for hypothesis 4. Concerning hypothesis 5, studies [57,63] reveal that, in OOHC, the buzzwords organic, regional and sustainable used for ingredients create profitable sales data when used in communications to end consumers. Studies show [64] that the majority of consumers do not distinguish between different sustainability labelling formats. Hence, it is an open question whether DNLs are accepted differently by consumers based on the sustainability dimension highlighted (regional, seasonal, etc.).

### 3.2. Study II—Design

Study II, a randomized choice experiment with factorial design, tested the best DNLs for each of the target dishes compared to the DE in a university canteen setting (*n* = 420). The analysis of this survey provided insights into the single effects of DNLs, single effects of the DE, and the combination of both.

**Figure 1:**
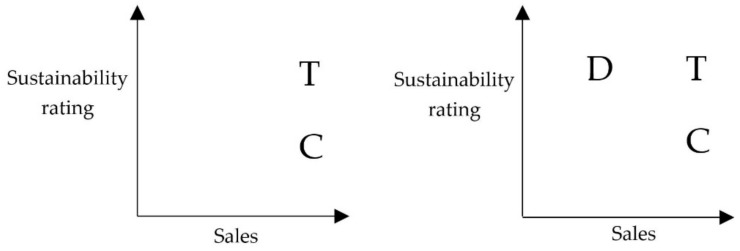
Exemplary illustration of the implementation of the decoy dishes. Note: Left: Without decoy dish/attraction effect, Right: With decoy dish/attraction effect. T = target dish, C = competitor dish, D = decoy dish. The figure based on [50].

The decoy dishes were intended to be less attractive to consumers. Attractiveness was assessed by using the proxy sales data as indicated by our experts (see Figure 1). The attractiveness had been assessed by nutrition experts with theoretical and practical backgrounds as well as using data from chefs and the kitchen directors at OOHC canteens.

The first of the decoy dishes mentioned in Table 5 was a carrot lasagne. This was inferior to the target vegetable lasagne, since the variety of ingredients was decreased to only one vegetable, the carrot. Experts have stated that this reduced variety negatively influences sales. The experts created the composition of the fish stew and noodles with pesto decoy dishes according to past sales data. Since the labels of these decoy dishes offer less information about the ingredients compared to the respective target dishes, sales data were lower. Chicken steak with celery puree was meant to be inferior due to the sales experience with celery puree in Germany (see Table 5).

**Figure 2:**
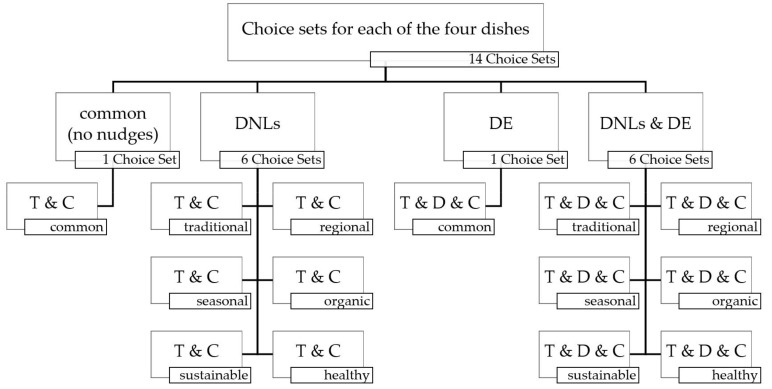
Factorial design to test the combination of DNLs and DE. Note: T = Target Dish (with DNLs), D = Decoy Dish, C = Competitor Dish.

To test the five hypotheses, a complete factorial design to evaluate all variants and combinations of the nudges was used. For the DNLs, all six different name categories with their best descriptive names, determined in study I, were used, resulting in 14 choice sets for each of the four dishes and 56 choice sets overall (see Figure 2).

**Figure A1:**
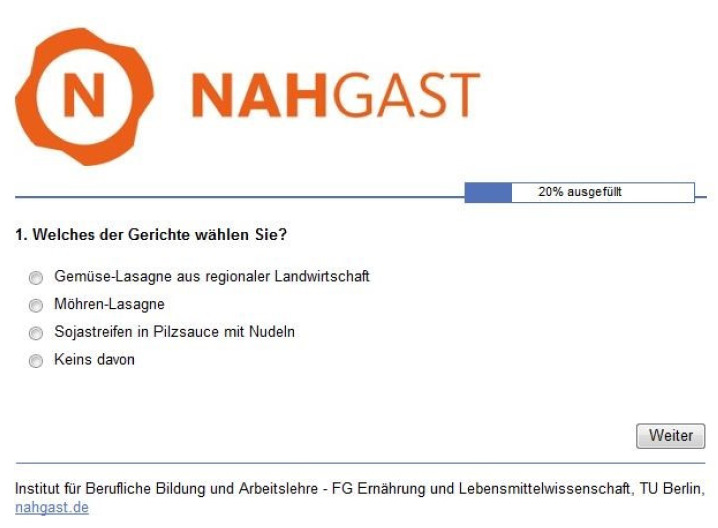
Exemplary choice set of Study II. Translation: Which of the dishes do you choose? Vegetable lasagne from regional agriculture vs. carrot lasagne vs. soy strips with noodles in mushroom sauce vs. none of them.

To avoid priming effects within one dish variant with different nudges, each survey participant received only one choice set of the 14 available for each dish. This resulted in four randomized choice sets to answer. To secure compliance with the factorial design, the 14 choice sets were computer-generated and drawn out of urns, one urn for each dish, without putting them back into the urn. This procedure was repeated after every 14 respondents. In addition to the food choice variants offered in the questionnaire, the interviewees were given the possibility to choose the "opt-out option". For an exemplary choice set of the survey, see Figure A1 in the Appendix A.

Study II was carried out in several university canteens at two universities in Berlin via personal interview (on a tablet computer) in September and October 2016. A total of 420 people who had lunch in these canteens took part in the survey. These 420 people were personally interviewed inside the dining area after eating their lunch. The survey took about 2 min to complete. A small pre-test revealed that the guests were very reluctant to take part in the survey before eating their lunch and before visiting the university canteens due to time pressure, stress and hunger. This attempt was therefore omitted due to excessive time expenditure by interviewers and canteen visitors, some of whom felt harassed. All respondents completed the survey entirely.

To test the hypotheses, we used a linear regression in Stata. We used the voting scores of the target dishes (calculated in percentages) as the dependent variable. As independent factor variables (where each level of group is included as a separate covariate/dummy [65]), we used the dish variant (1 = vegetable lasagne, 2 = breaded fish with fried potatoes, 3 = spaghetti with rocket pesto, 4 = chicken steak with tagliatelle) and the nudging variant (1 = DNLs and DE, 2 = DNLs, 3 = DE, 4 = common) for hypotheses 1–4. For hypothesis 5, we used the dish variant and the descriptive name label variants (1 = traditional, 2 = regional, 3 = seasonal, 4 = organic, 5 = sustainable, 6 = healthy, 7 = common) to reveal possible differences between each of the single variants. Our results of the combination of DNLs and the DE (where the DE most likely had a strong negative influence) would influence the results used to answer hypothesis 5, if calculated in a joint model (14 choice sets per dish, see Figure 2). Therefore, we calculated a separate model for hypothesis 5 with a subset of data, where only pure DNLs and the base measurement without nudges were included (seven choice sets per dish, see Figure 2). The assumption checks of our two linear regression models were without conspicuities (no multicollinearity—mean variance inflation factors 2.39 and 1.64 [66]; normal distribution—skewness and kurtosis test (described by D’Agostino et al. [67] with the adjustment made by Royston [68]) *p*-value *p* = 0.8950 and *p* = 0.7817; no heteroscedasticity—White test *p*-values *p* = 0.116 and *p* = 0.061 [69]; no autocorrelation—Durbin–Watson-test d(7.56) = 2.08 and d(10.28) = 1.28 [70]).

### 3.3. Study II—Results

**Figure 3:**
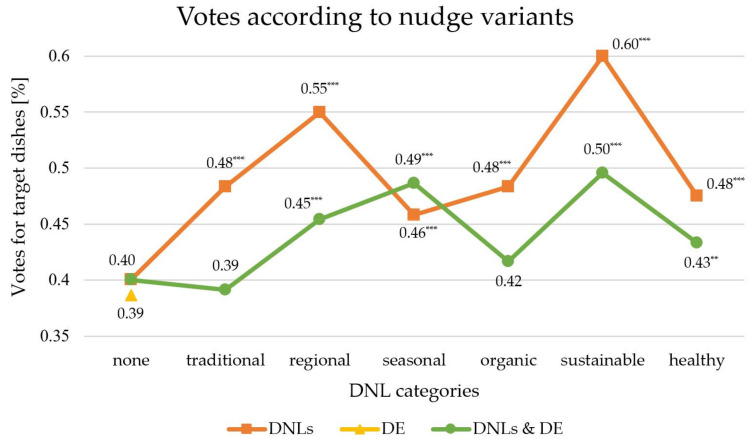
Descriptive results of Study II—DNLs, DE and DNLs and DE. Note: *** *p* < 0.001, ** *p* < 0.01, * *p* < 0.05 (pairwise *t*-test). The single decoy effect (DE) has only one data point in the “none” category.

Over the entire survey, the common target dishes were chosen by participants about 40.0 percent of the time (see Figure 3). On average, target dishes with DNLs were about ten percent more favoured at 50.8 percent and were still ahead of their competitor dishes.

Adding a decoy to the choice set decreased the choice frequency of the target dish. The target food with the DE was less popular than its common variants and led to a drop in overall votes. If the target dishes were combined with the DNLs and the DE, the result was a 44.6 percent choice frequency and an overall increase in votes compared to common target dishes. As seen in Figure 3, the strong effect of DNLs becomes clear in comparison to the other two nudging variants—the DE and the combination of both. By using the decoy effect, even the overall votes for target foods decreased and clearly improved in combination with DNLs, but this was solely due to the effect of the DNLs. From a descriptive point of view, the observations show that only the DNLs had a positive influence on choice behaviour, especially regional (55 percent) and sustainable (60 percent) DNLs. Figure 3 shows the difference in choice frequencies between pure DNLs, the DE and the combination of both for each DNL category.

Table 6 displays the results of the linear regression for hypotheses 1–4. The test for a positive significant difference in respondents’ voting behaviour between DNLs and their common dish variants (β-coefficient β = 0.108; *p*-value *p* ≤ 0.001), supports hypothesis 1. The use of descriptive names leads to significantly higher choices of the target dishes among the respondents.

Hypothesis 2, the test of the DE, must be rejected (β = −0.012; *p* = 0.266). Target dishes with a decoy were not chosen significantly more frequently than the target dishes without a decoy but even were “cannibalized” by their decoy dishes.

Supporting hypothesis 3, the combination of DNLs and the DE (*β* = 0.046; *p* ≤ 0.001) did lead to significantly higher choice results for the target dishes with nudges in comparison to their common dish counterparts without.

As a result of the first three hypotheses, hypothesis 4 must also be rejected. The pure DE did not show any significant difference and the combination of DNLs and the DE had a lower impact on the target dishes than pure DNLs, as seen by the β-coefficients. The DNLs therefore had the highest positive influence on sustainable meal choices. Within study II, the combination of the two nudges is inferior to the use of pure DNLs.

Table 7 displays the results of the linear regression for hypothesis 5. The test of significant differences in the effectiveness of DNL categories supports hypothesis 5. All DNL categories had a significant positive influence on the meal choice of the target dishes compared to their common counterparts. Indeed, for the categories seasonal (β = 0.058; *p* ≤ 0.001), healthy (β = 0.075; *p* ≤ 0.001), organic (β = 0.083; *p* ≤ 0.001) and traditional (β = 0.083; *p* ≤ 0.001) there were significant differences in choice compared to target dishes without DNLs. However, for the regional name category (β = 0.150; *p* ≤ 0.001), as well as for the sustainable name category (β = 0.200; *p* ≤ 0.001), these differences were considerably stronger. A pairwise *t*-test underlined the strong marketing effect of regional and sustainable DNLs, with their increase in choices of up to 20 percent compared to their common dish variants without the use of nudges. Table 8 illustrates the pairwise *t*-test, in which the use of regional and especially sustainable DNLs scored significantly better than the other DNL options.

Another important factor besides the evaluation of our five hypotheses was the influence of the respective target dishes on choice behaviour. As can be seen in both regression models (see Table 6 and Table 7), lasagne and especially spaghetti worked better with their attached nudges compared to the chicken steak or the breaded fish. Therefore, it is important to not only check for nudging variants but also for the specific dish and nudge combination.

## 4. Study III

### 4.1. Study III—Design

During four weeks in October and November 2016, study III was conducted in a business canteen with four selected DNLs and DE choice sets from study II (see Table 9). One of these choice sets was put on tablet computers in the dining area of the business canteen each week. These tablet computers had already been used by the business canteen to select the special dish offered the following week, with no further questions asked about sociodemographic characteristics. With this tool, the respondents chose their preferred meal spontaneously without knowing about the ongoing study. During the four weeks, a total of 820 visitors took part in the tablet survey (for detailed respondent numbers per week, see Table 10). Due to delivery difficulties, in week 4 not all the ingredients of the dish but only tomatoes could be locally sourced for the business canteen. With a Mann–Whitney U Test [71] we checked for choice differences between the university canteen (study II) and the business canteen (study III) settings.

### 4.2. Study III—Results

Table 9 displays the voting results of the business canteen guests. During week 1 (vegetable lasagne: 51.1%), week 2 (breaded fish: 47.9%) and week 4 (chicken steak: 46.1%) of our survey, the target dishes prevailed, while in week 3 the competitor dish was preferred (mustard eggs: 43.5%). Table 10 reveals that for vegetable lasagne (z = −0.286; *p* = 0.775), there was no change in choice of the target food between settings. For breaded fish (z = 1.647; *p* = 0.100) and chicken steak (z = 1.806; *p* = 0.071) there are significant tendencies. Only for spaghetti (z = 2.740; *p* = 0.006), significant differences between the two OOHC settings could be recorded. With only one significant change out of four target dishes, assumptions cannot be made as to the direct transferability of results from the university canteen in study II to the company canteen. As seen in previous studies, the results of nudging interventions are not only influenced by the type of nudge or the type of setting but also by other variables, e.g., the offered target dishes (see Study II), or the weather or the day of eating [34].

## 5. Discussion and Conclusions

The present research provides insights into the ability to increase the choice of sustainable dishes by combining nudges. Two nudges were examined: the use of different descriptive name labels (DNLs) that, according to the previous literature [41,42,43,44,45], increase choice frequency compared to respectively named dishes without DNLs, and the decoy effect [35,36,37,38,40,50,62], which in non−food contexts has been able to influence consumer choices in an intended direction. The feasibility and application of both nudges were evaluated in two different OOHC settings. Our studies reveal that descriptive name labels were able to influence choices positively insofar as they resulted in the choice of a more sustainable dish. Significant differences between the various tested descriptions indicate that the story told to guests/consumers matters. For example, a meal labelled as ‘healthy’ is less attractive than a ‘regional’ meal. Overall, it became obvious that the general term, ‘sustainable’, was most successful in influencing choices. Therefore, we conclude that consumers differentiate between the various sustainability dimensions but appreciate sustainable meals in general. Since we did not ask guests about their understanding of sustainability as a general concept or how it relates to food production and consumption, we cannot elaborate on the reasons for this finding.

On the basis of our results, we cannot recommend the joint use of DNLs and the DE as a tool for boosting sustainable food choices. We reject hypothesis 4, as the DE had no significant impact on choice frequency of target dishes and, in fact, the inclusion of the pure DE lowered the overall choice frequency of target dishes (hypothesis 2). It is clearly superior to apply single DNLs, especially the two most effective DNLs (‘regional’; ‘sustainable’) as identified in our work (compare hypothesis 5). When comparing the best DNLs from the six name categories, all variants were able to significantly and positively influence respondents’ choices. Study participants preferred the descriptions ‘regional’ (choice increase of around 15%) and ‘sustainable’ (choice increase of about 20%). It should be noted, however, that only one possible DNL per category was tested in this study. For the regional DNL category, this was the DNL “from regional agriculture/fisheries” and for the sustainable name category the tested DNL was “from sustainable agriculture/fisheries”.

The negative effect of applying the DE to influence dish choice stands in contrast to the previous non-food literature [35,36,37,38,62]. Regarding the food sector and especially OOHC, a recently published online choice experiment study focused on the addition of a price decoy to a menu with the aim of promoting vegetarian target options. In this study by Attwood et al. [72], the vegetarian decoy was more expensive (up to 30%) than the less expensive vegetarian target dish. Results revealed that the addition of the decoy dish did not lead to a higher selection rate of the vegetarian target dish compared to the meat competitor option. One possible reason for the failure of the nudge in that study was the use of a price decoy strategy. Alternative decoy strategies which do not focus on price but on other attributes (e.g., menu description or caloric content) might work when displayed to consumers [72]. Even though sales data and sustainability ratings instead of price differences were used to define the decoy dishes in our study, the DE nudge did not work as expected. A possible reason is that consumers were not aware of e.g. the different sustainability ratings of the dishes as they were not indicated besides the dishes name. Another possible explanation for the non-significant finding of the decoy effect is that the meals assigned as competitor dishes, decoy dishes and target dishes by the project team and the experts might not have been perceived in these roles by canteen guests. For example, the experts had not expected canteen guests to prefer the decoy carrot lasagne over the target vegetable lasagne, which offered a larger variety of vegetables for the same price. The experts thought that variety seeking consumers would prefer a combination of vegetables over just one variety. Hereby, the experts might have overlooked that the complete information on vegetable ingredients in the case of the carrot lasagne simplified consumers’ choice compared to a vegetable lasagne. The unknown combination of various vegetables might have contained some ingredients consumers might not like or hesitate to eat from, e.g., a health perspective. However, we did not employ questionnaires and so were unable to confirm why consumers’ evaluations differed from the experts’ expectations. Hence, the careful selection and grouping of the target and the competitor dishes is of fundamental relevance for the study results. The challenge researchers and practitioners face when designing study designs and menus is that, to date, meals and dishes have never been categorized as target or competitor dishes. Our decision to classify dishes in the competitor/target group was based on the experts’ opinion and not on a systematic assessment of similarities and differences between dishes. Identifying consumers’ perception of perceived similarity of various product characteristics and attribute levels is thus one important step future research has to take.

Based on these results, further research should also uncover and explore possible applications of the decoy effect in the field of food choice. In addition, the discussion of possible moderators of decoy effects and possible decoys should be continued. Such debates should take place in specialized contexts, e.g., in the context of sustainable food consumption or OOHC settings, since preferences often differ between several choice environments [34,73], as seen in study III.

It is important that nudges be applied situation-specifically. It is also advisable to monitor changes in guest behaviour when using new or unknown nudges in a specific setting [42,74]. It may happen, as study II suggests, that nudges do not always work in the desired direction, especially when several nudges are combined, and varying target dishes are used. Future research uncovering the interaction of nudges and different dish categories in OOHC is therefore recommended.

The preferences of OOHC consumers towards more regional and sustainable DNLs point to the wish for more regional food and overall sustainable ingredients in the product ranges of OOHC, which also corresponds to today’s trend in Germany [57]. Needless to say, the respective food quality of products advertised with DNLs should be maintained, as a decline in quality would cause consumer expectations to inflate [41,42].

Catering managers, canteen and restaurant owners should follow this regional and sustainable trend, with the use of behavioural insights, nudges and further interventional approaches to promote relevant products and contribute to the transformation towards a more sustainable economy.

## Figures and Tables

**Table 1:** Overview of the three studies.

	Study I	Study II	Study III
Content	Determine the best Descriptive name label (DNL) wording	Test of the isolated nudges decoy effect (DE) and DNLs, as well as the combination DNLs and DE	Test of the nudge combination DNLs and DE
Method	Focus group; Choice experiment; Best choice	Choice experiment; Linear regression	Choice experiment; U-Test (Mann/Whitney)
Sample	Students; *n* = 100	University canteen; *n* = 420	Business canteen; *n* = 820

**Table 2:** Target and Competitor Dishes.

Target Dishes	Competitor Dishes
Vegetable lasagne	Soy strips with noodles in mushroom sauce
Breaded fish with fried potatoes	Escalope chasseur with French fries
Spaghetti with rocket pesto	Mustard eggs with mashed potatoes
Chicken steak with tagliatelle	Spaghetti Bolognese

**Table 3:** Desirable and undesirable sustainability aspects in out-of-home catering (OOHC) (focus group).

Desirable	Undesirable	Ambivalent
Free of… Regional Seasonal Pesticide-free Freshness Animal Welfare Hygiene	CO2/Carbon Footprint Ecological Footprint Additives Percentage of animal products Resource input	Organic Sugar Nutritional information Fair trade Free of genetic modifications Portion size

The table, based on [55].

**Table 4:** Overview of the pre-tested descriptive name labels (DNL) of Study I (*n* = 100).

Overview of The Descriptive Name Labels (DNL) of Study I			
Traditional	[%]	Regional	[%]
(Dish), traditional style	63.5	(Dish) from regional agriculture	28.5
Grandma’s (Dish)	16.5	(Dish) from the region	21.0
(Dish) according to grandma’s secret recipe	11.5	(Dish) from region XYZ	18.5
(Dish), the ancient way	5.0	(Dish) from regional production	17.5
(Dish) according to Aunt Martha’s secret recipe	2.5	Region XYZ (Dish)	14.0
Missing	1.0	Missing	0.5
Seasonal	[%]	Organic	[%]
(Dish) with seasonal ingredients	64.0	Organic (Dish)	35.5
(Dish) from seasonal agriculture	22.0	(Dish) from organic agriculture	16.0
(Dish) from seasonal production	14.0	(Dish) from organic production	15.5
Missing	0.0	(Dish) from ecological production	10.5
		(Dish) produced according to ecological standards	7.0
		(Dish) from ecological agriculture	7.0
		(Dish) from eco-friendly agriculture	5.0
		(Dish) from eco-friendly production	3.5
		Missing	0.0
Sustainable	[%]	Healthy	[%]
(Dish) from sustainable agriculture	22.5	(Dish) low-energy prepared	24.5
(Dish) from sustainable production	18.5	Low energy (Dish)	19.0
Fair trade (Dish)	17.0	Light (Dish)	17.0
(Dish) from fair production	8.5	(Dish) for light pleasure	17.0
(Dish) from fair trade agriculture	6.5	(Dish) with few calories	6.0
Fairly traded (Dish)	6.5	Calorie-reduced (Dish)	5.0
(Dish) from fair trade production	6.0	(Dish) for light nutrition	5.0
(Dish) from fair agriculture	5.0	(Dish) with reduced calories	1.0
(Dish) produced according to social standards	3.5	Missing	5.5
(Dish) produced according to ethical standards	1.0		
Missing	5.0		

Note: For simplicity, this table bears the neutral “Dish” designation. In this evaluation, however, all votes across all five dishes are included. DNLs translated from German.

**Table 5:** Target, Decoy and Competitor Dishes.

Target Dishes	Decoy Dishes	Competitor Dishes
Vegetable lasagne	Carrot lasagne	Soy strips with noodles in mushroom sauce
Breaded fish with fried potatoes	Fish stew	Escalope chasseur with French fries
Spaghetti with rocket pesto	Noodles with pesto	Mustard eggs with mashed potatoes
Chicken steak with tagliatelle	Chicken steak with celery puree	Spaghetti Bolognese

**Table 6:** Linear Regression (dependent variable (DV): Target dish selection, independent variable (IV): Nudge variant, Dish variant).

Target	Coefficient	Standard Error	*t*	P > *t*
Nudge				
DNL	0.108	0.009	12.69	0.000
DNL and DE	0.046	0.009	5.44	0.000
DE	−0.012	0.011	−1.11	0.266
common	0	(base)		
Dish				
Spaghetti with rocket pesto	0.198	0.006	33.18	0.000
Vegetable lasagne	0.169	0.006	28.41	0.000
Chicken steak with tagliatelle	0.067	0.006	11.21	0.000
Breaded fish with fried potatoes	0	(base)		
constant	0.292	0.009	33.61	0.000
Number of observations	1680			
*R*-squared	0.5175			

**Table 7:** Linear Regression (DV: Target dish selection, IV: DNL variant, Dish variant).

Target	Coef.	Std. Err.	*t*	P > *t*
DNL				
sustainable	0.200	0.009	21.70	0.000
regional	0.150	0.009	16.24	0.000
traditional	0.083	0.009	9.01	0.000
organic	0.083	0.009	9.01	0.000
healthy	0.075	0.009	8.12	0.000
seasonal	0.058	0.009	6.30	0.000
common	0	(base)		
Dish				
Spaghetti with rocket pesto	0.248	0.007	35.59	0.000
Vegetable lasagne	0.167	0.007	23.95	0.000
Chicken steak with tagliatelle	0.091	0.007	13.01	0.000
Breaded fish with fried potatoes	0	(base)		
constant	0.274	0.008	35.21	0.000
Number of observations	840			
*R*-squared	0.7043			

**Table 8:** Pairwise *t*-test between the pure descriptive name labels (DNL) on choice behaviour.

DNL	Contrast	Std. Err.	*t*	P > *t*
regional vs. traditional	0.067	0.015	4.43	0.000
seasonal vs. traditional	−0.025	0.015	−1.66	0.096
organic vs. traditional	0.00	0.015	0.00	1.000
sustainable vs. traditional	0.117	0.015	7.77	0.000
healthy vs. traditional	−0.008	0.015	−0.55	0.583
common vs. traditional	−0.083	0.015	−5.53	0.000
seasonal vs. regional	−0.092	0.015	−6.09	0.000
organic vs. regional	−0.067	0.015	−4.43	0.000
sustainable vs. regional	0.050	0.015	3.35	0.001
healthy vs. regional	−0.075	0.015	−4.98	0.000
common vs. regional	−0.150	0.015	−9.95	0.000
organic vs. seasonal	0.025	0.015	1.66	0.096
sustainable vs. seasonal	0.142	0.015	9.44	0.000
healthy vs. seasonal	0.017	0.015	1.12	0.265
common vs. seasonal	−0.058	0.015	−3.86	0.000
sustainable vs. organic	0.117	0.015	7.77	0.000
healthy vs. organic	−0.008	0.015	−0.55	0.583
common vs. organic	−0.083	0.015	−5.53	0.000
healthy vs. sustainable	−0.125	0.015	−8.32	0.000
common vs. sustainable	−0.200	0.015	−13.30	0.000
common vs. healthy	−0.075	0.015	−4.98	0.000

**Table 9:** Results of the DNLs and DE choice experiment in the business canteen.

Results of the DNLs and DE Choice Experiment in the Business Canteen			
	Target Dishes	Decoy Dishes	Competitor Dishes
Week 1	Vegetable lasagne traditional style	Carrot lasagne	Soy strips with noodles in mushroom sauce
	51.1%	30.1%	18.8%
Week 2	Breaded fish from sustainable fisheries with fried potatoes	Fish stew	Escalope chasseur with French fries
	47.9%	10.6%	41.5%
Week 3	Spaghetti with rocket pesto with seasonal ingredients	Noodles with pesto	Mustard eggs with mashed potatoes
	34.1%	22.4%	43.5%
Week 4	Chicken steak with tagliatelle and tomatoes from regional agriculture	Chicken steak with celery puree	Spaghetti Bolognese
	46.1%	19.7%	34.2%

**Table 10:** Mann−Whitney U Test for OOHC setting comparison of the combined DNLs and DE nudges.

Target Dishes	OOHC Setting	Number of Observations	Rank Sum	z	Asymp. Sig. (2-Tailed)
Vegetable lasagne traditional style	University	25	1935.5	−0.286	0.775
	Business	133	10625.5		
Breaded fish from sustainable fisheries with fried potatoes	University	21	3174.5	1.647	0.100
	Business	236	29978.5		
Spaghetti with rocket pesto with seasonal ingredients	University	26	4046.0	2.740	0.006
	Business	223	27079.0		
Chicken steak with tagliatelle (and tomatoes) from regional agriculture	University	21	3118.5	1.806	0.071
	Business	228	28006.5
